# Pretreatment Axillary Nodal Volume as a Prognostic Factor for Breast Cancer

**DOI:** 10.1155/tbj/1823771

**Published:** 2025-04-23

**Authors:** Yuri Jeong, Jung Hoon Kim, Su Ssan Kim, Jinhong Jung, Ji Hyeon Joo, Hwa Jung Kim, Hak Hee Kim, Joo Hee Cha, Hee Jung Shin, Seung Do Ahn

**Affiliations:** ^1^Department of Radiation Oncology, Wonkwang University Hospital, Wonkwang University School of Medicine, Iksan, Republic of Korea; ^2^Department of Radiation Oncology, Konyang University Hospital, Daejeon, Republic of Korea; ^3^Department of Radiation Oncology, Asan Medical Center, University of Ulsan College of Medicine, Seoul, Republic of Korea; ^4^Department of Radiation Oncology, Pusan National University Yangsan Hospital, Pusan National University School of Medicine, Yangsan, Republic of Korea; ^5^Department of Clinical Epidemiology and Biostatistics, Asan Medical Center, University of Ulsan College of Medicine, Seoul, Republic of Korea; ^6^Department of Radiology, Asan Medical Center, University of Ulsan College of Medicine, Seoul, Republic of Korea

**Keywords:** axilla, breast neoplasm, lymph nodes, prognosis, survival

## Abstract

**Background and Objectives:** We evaluated the prognostic value of pretreatment axillary nodal volume in breast cancer patients treated with neoadjuvant systemic therapy.

**Methods:** We retrospectively reviewed 302 breast cancer patients with biopsy-proven axillary LN involvement who received neoadjuvant systemic therapy. Axillary nodal volumes were obtained from pretreatment magnetic resonance imaging. Univariate and multivariate analyses for disease-free survival (DFS) and overall survival (OS) rates were conducted.

**Results:** The median follow-up period was 57.0 months, and 5-year DFS and OS rates were 81.6% and 91.9%, respectively. Pretreatment axillary nodal volume ranged from 0.2 mL to 134.2 mL, and the first tertile (2.6 mL) and fifth quintile (12.0 mL) were chosen as the optimal cutoff points for survival outcomes. In the multivariate analysis, nodal volume (< 2.6 mL vs. 2.6–12.0 mL vs. ≥ 12.0 mL) was a significant prognostic factor for DFS (5-year DFS, 90.1% vs. 79.6% vs. 72.2%) and OS (5-year OS, 97.9% vs. 90.9% vs. 84.2%), whereas the *N* stage was not.

**Conclusions:** In breast cancer patients treated with neoadjuvant systemic therapy, larger pretreatment axillary nodal volume was associated with poor survival outcomes.

## 1. Introduction

Breast cancer patients with axillary lymph node (LN) involvement often receive neoadjuvant systemic to downstage both the primary tumor and axillary LNs. Although the frequency of downstaging, including pathologic complete response (pCR), varies by molecular subtype [1, 2], ypN stage, particularly axillary pCR, has been reported as an important prognostic factor [3–8]. Studies have shown that patients achieving axillary pCR can have favorable oncologic outcomes even without axillary LN dissection [9–12]. Based on these findings, recent guidelines recommend that biopsy-proven clinically node-positive patients who achieve axillary pCR after neoadjuvant systemic therapy can omit further axillary surgery following sentinel node biopsy [12–14].

While ypN stage has been studied extensively, research on the prognostic significance of pretreatment clinical N stage in patients receiving neoadjuvant systemic therapy has been limited [15]. In axillary LN-positive breast cancer, the clinical N stage is determined by the anatomic level of involved axillary LNs (levels I, II, or III) and their fixation [16]. However, the assessment for the level of axillary LNs involved and their fixation is highly dependent on the imaging modality and the physician's physical examination. Despite the limitation, current guidelines recommend adjuvant radiotherapy depending on the maximal stage among the pathologic and clinical stage, regardless of response after neoadjuvant systemic therapy [13, 17, 18]. In patients with cT1-2 breast cancer patients who received neoadjuvant systemic therapy, clinical *N* stage remains important for the decisions related to postmastectomy radiotherapy or the addition of regional nodal irradiation to breast radiotherapy. Therefore, new strategies to improve the objectiveness and clinical significance for clinical *N* stage are needed.

Recently, magnetic resonance imaging (MRI) has been frequently used for initial workup in patients with axillary LN-positive breast cancer, and the objective assessment of axillary nodal volume has become possible. However, little data have been published on the prognostic value of the pretreatment tumor burden of axillary LNs [19–21]. In the present study, we evaluated the prognostic value of axillary nodal volume using pretreatment MRI in axillary LN-positive breast cancer.

## 2. Materials and Methods

### 2.1. Patients

We retrospectively reviewed the records of 428 patients who were diagnosed with cT1-2N1-3a breast cancer between October 2007 and December 2013 and received neoadjuvant systemic therapy followed by surgery at Asan Medical Center. Of these patients, 126 were excluded for the following reasons: (1) bilateral breast cancer (*n* = 2); (2) a history of previous malignancy other than thyroid cancer and skin cancer (*n* = 1); (3) a short follow-up period of less than 6 months after surgery (*n* = 8); (4) no information about adjuvant radiotherapy (*n* = 40); (5) no pathologic confirmation of axillary LN involvement (*n* = 17); and (6) no pretreatment MRI evaluation (*n* = 58). The remaining 302 patients with cT1-2N1-3a breast cancer who had biopsy-proven axillary LN involvement and underwent pretreatment MRI were included in the present study. This study was approved by the Institutional Review Board at Asan Medical Center.

### 2.2. Staging and Nodal Volume

The clinical stage was determined according to the eighth edition of the American Joint Committee on Cancer staging system. Chest CT and bone scans were routinely performed to rule out metastatic disease. In our study, PET/CT scans were additionally conducted in 276 (91.4%) of 302 patients. The anatomic level of involved axillary LNs (I, II, or III) and their fixation were determined by MRI and physical examination, respectively. For patients with involvement of multiple levels of axillary LNs, the highest level of involvement was used for classification (e.g., involvement of both Levels I and II was classified as Level II). The status of the estrogen receptor (ER), progesterone receptor (PR), and human epidermal growth factor Receptor 2 (HER2) was examined using immunohistochemistry, and in situ hybridization was also used when HER2 status was equivocal. To evaluate axillary nodal volume, we imported the postcontrast-enhanced T1-weighted axial images from pretreatment MRI into the eclipse treatment planning system (Varian, Palo Alto, CA), which is a commercial software for radiotherapy planning. The axillary LNs with the shortest diameter of ≥ 0.5 cm in the axial images were contoured by a board-certified radiation oncologist (Y.J.), who was blind to the clinical stage and survival outcomes, and total nodal volume was automatically calculated using eclipse treatment planning system.

### 2.3. Statistics

Disease-free survival (DFS) and overall survival (OS) rates were estimated from the date of the start of neoadjuvant systemic therapy to the date of locoregional recurrence, distant metastasis, death from any cause, or last follow-up and to the date of death from any cause or last follow-up, respectively, using the Kaplan–Meier method. Univariate and multivariate analyses using the Cox proportional hazards model were conducted to identify associations of variables with DFS and OS. The variables included in the univariate analysis were age, cT, N stage, level of axillary LNs, nodal volume, histologic grade, hormone receptor status, HER2 status, Ki-67, breast surgery, axillary surgery, ypT, N stage, adjuvant radiotherapy, adjuvant chemotherapy, adjuvant hormone therapy, and adjuvant targeted therapy. Variables with *p* values of < 0.1 were included in the multivariate analysis, and backward elimination of Cox's regression was used. In addition, clinically relevant variables, such as cN stage and ypN stage, were also included regardless of their *p* value in the univariate analysis, to account for their potential prognostic significance. The optimal cutoff points for axillary nodal volume were chosen from among the quantile-based cutoffs (median, tertiles, quartiles, and quintiles), selecting those with the smallest *p* value for DFS rate in the log-rank statistics. The association between axillary nodal volume and the other variables was analyzed using Kruskal–Wallis test and Mann–Whitney *U* test. All statistical tests were two-sided and performed at a 5% level of significance using SPSS (Version 21.0; SPSS Inc., Chicago, IL, USA).

## 3. Results

Patient characteristics are summarized in Table 1. The median age of the patients was 47 years (range, 26–75 years), and 248 (82.1%), 26 (8.6%), and 28 (9.3%) patients were determined to be in cN1, cN2a, and cN3a stages, respectively. The level of axillary LN involved was up to Levels I, II, and III in 176 (58.3%), 98 (32.5%), and 28 (9.3%) patients, respectively. The 179 (59.3%) patients were ER (+) and/or PR (+), and 103 (34.1%) were HER2 (+). In terms of neoadjuvant systemic therapy, chemotherapy and hormone therapy were performed on 277 (91.7%) and 25 (8.3%) patients, respectively, and targeted therapy was combined in 19 (6.3%) patients.

### 3.1. Survival Rates Depending on Axillary Staging and Nodal Volume

**Figure 1:**
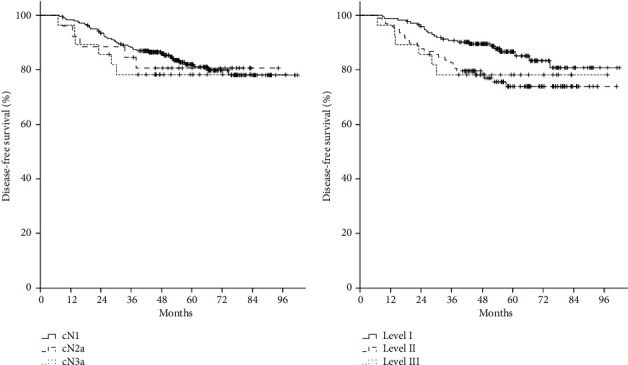
Disease-free survival rate by clinical *N* stage (a) and the level of involved axillary LNs (b).

**Figure 2:**
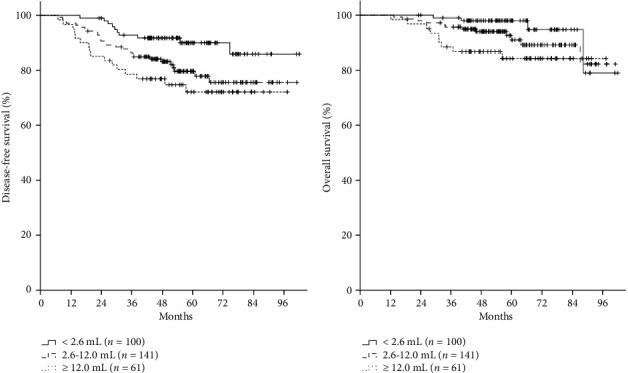
Disease-free survival (a) and overall survival (b) rates depending on the cutoffs for the first tertile and fifth quintile of pretreatment axillary nodal volume.

The follow-up period was median 57.0 months (range, 12.1–102.0 months). The patterns of failure were distant metastasis, locoregional recurrence, and both in 20 (6.6%), 9 (3.0%), and 24 (7.9%) patients, respectively. The 5-year DFS rate was 81.6%. The difference in 5-year DFS was not statistically significant between clinical *N* stages (cN1 vs. cN2a vs. cN3a, 82% vs. 80.6% vs. 78.3%, *p*=0.779) and the level of axillary LNs involved (Level I vs. Level II vs. Level III, 86.7% vs. 74.0% vs. 78.3%, *p*=0.080; Figures 1(a) and 1(b)). Pretreatment axillary nodal volumes ranged from 0.2 mL to 134.2 mL, and the quantile-based cutoffs (median, tertiles, quartiles, and quintiles) were as follows: median, 4.1 mL; tertiles, 2.6 mL and 7.0 mL; quartiles, 2.0 mL, 4.1 mL, and 9.6 mL; quintiles, 1.7 mL, 3.1 mL, 5.7 mL, and 12.0 mL. The 5-year DFS rates for these cutoffs were as follows (Supporting figure 1(a)–1(d)): median, 86.5% vs. 76.8% (*p*=0.044); tertiles, 90.1% vs. 77.9% vs. 76.3% (*p*=0.035); quartiles, 87.9% vs. 84.8% vs. 80.1% vs. 73.5% (*p*=0.166); and quintiles, 89.8% vs. 86.7% vs. 77.4% vs. 81.0% vs. 72.2% (*p*=0.170). We chose 2.6 mL and 12.0 mL, which were the cutoffs for the first tertile and fifth quintile, as the optimal cutoff points, and classified pretreatment nodal volume into three groups: low (< 2.6 mL), intermediate (2.6–12.0 mL), and high (≥ 12.0 mL). The 5-year DFS rates were 90.1%, 79.6%, and 72.2% in patients with low, intermediate, and high nodal volumes, respectively (*p*=0.023, Figure 2(a)). The 5-year OS rate was 91.9% for all patients, and 97.9%, 90.9%, and 84.2% for patients with low, intermediate, and high nodal volumes, respectively (*p*=0.072, Figure 2(b)).

### 3.2. Prognostic Factors for Survival Rates

The results of the univariate and multivariate analyses are shown in Table 2. For DFS, nodal volume, age, ypT stage, adjuvant radiotherapy, and adjuvant hormone therapy were significant prognostic factors in the multivariate analysis, but clinical N stage, the level of involved axillary LNs, and ypN stage were not. The hazard ratios (95% confidence interval [CI], *p* value) of intermediate and high nodal volumes were 2.132 (1.025–4.434, 0.043) and 3.098 (1.323–7.257, 0.009), respectively.

For OS, nodal volume was the only significant prognostic factor (*p*=0.028) for OS, and the hazard ratios (95% CI, *p*-value) of intermediate and high nodal volumes were 2.421 (0.655–8.943, 0.185) and 5.408 (1.463–19.993, 0.011), respectively.

### 3.3. Relationship Between Nodal Volume and Clinicopathologic Factors

The median nodal volume increased with clinical N stage: cN1 vs. cN2a vs. cN3a = 3.4 mL (range, 0.2–37.1 mL) vs. 11.4 mL (2.3–60.3 mL) vs. 17.3 mL (1.2–134.2 mL). Differences in nodal volume were statistically significant between cN1 and N2a (*p* < 0.001), and cN1 and N3a (*p* < 0.001) but not between cN2a and cN3a (*p* = 0.097). The nodal volume significantly differed between the levels of involved axillary LNs (*p* < 0.001), with the median nodal volume increasing with a rise in the level of involved axillary LNs: Level I vs. Level II vs. Level III = 2.9 mL (range, 0.2–37.1 mL) vs. 5.7 mL (range, 0.3–60.3 mL) vs. 17.3 mL (range, 1.2–134.2 mL). The median nodal volume in patients with ER (−) and PR (−) was 7.1 mL (range, 0.7–134.2 mL), which was significantly higher than that in patients with ER (+) and/or PR (+), who has a median of 3.2 mL (range, 0.2–60.3 mL).

### 3.4. Subgroup Analysis According to Hormone Receptor Status

**Figure 3:**
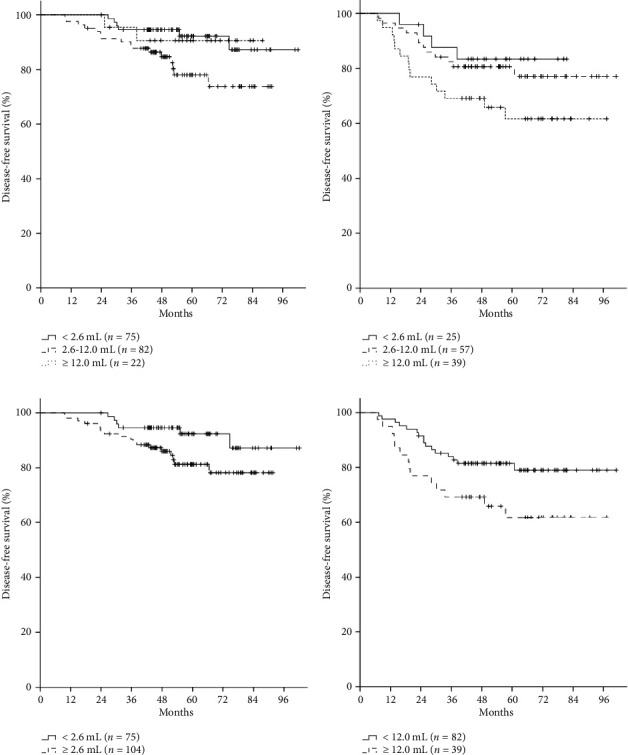
Disease-free survival (DFS) rate by axillary nodal volume in subgroups of hormone receptor status. (a) DFS rate by the cutoffs for the first tertile and fifth quintile of axillary nodal volume in patients with ER (+) and/or PR (+). (b) DFS rate by the cutoffs for the first tertile and fifth quintile of axillary nodal volume in patients with ER (−) and PR (−). (c) DFS rate by the cutoff for the first tertile of axillary nodal volume in patients with ER (+) and/or PR (+). (d) DFS rate by the cutoff for the fifth quintile of axillary nodal volume in patients with ER (−) and PR (−).

In patients with ER (+) and/or PR (+), the 5-year DFS rate for patients with low nodal volume was higher than that for patients with intermediate to high nodal volume (92.3% vs. 81.3%, *p*=0.059; Figures 3(a), 3(c)). In patients with ER (−) and PR (−), the 5-year DFS rate for patients with high nodal volume was lower than that in patients with low to intermediate nodal volume (81.5% vs. 61.7%, *p*=0.045; Figures 3(b) and 3(d)).

### 3.5. Subgroup Analysis According to ypN Stage

In patients with ypN0 stage, the 5-year DFS rates did not differ significantly based on nodal volume (87.1%, 82.2%, and 81.5% for low, intermediate, and high nodal volume, respectively; *p*=0.854) or cN stage (83.7%, 90.0%, and 76.9% for cN1, N2a, and N3a, respectively; *p*=0.719). In patients with ypN1-3a stage, the 5-year DFS rates were significantly different based on nodal volume (92.1%, 78.6%, and 64.5% for low, intermediate, and high nodal volume, respectively; *p*=0.008), while cN stage showed no significant differences (79.5%, 75.0%, and 80.0% for cN1, N2a, and N3a, respectively; *p*=0.753).

## 4. Discussion

Our study demonstrated that pretreatment axillary nodal volume is a significant prognostic factor for both DFS and OS in patients with axillary LN-positive breast cancer receiving neoadjuvant systemic therapy, while clinical N stage, level of involved axillary LNs, and ypN stage were not. Pretreatment nodal volume offers additional, objective insight into axillary tumor burden, addressing some of the limitations of clinical *N* stage assessment. Current guidelines highlight the role of axillary pCR in reducing the extent of axillary surgery, recommending the omission of axillary LN dissection in patients who achieve axillary pCR after neoadjuvant systemic therapy. These findings are consistent with recent trends in axillary cavity management, which favor de-escalate axillary surgery, in the upfront surgery approach [12, 13]. As less invasive surgery becomes more common in breast cancer management, integrating pretreatment nodal volume into clinical decision-making could improve patient stratification, particularly by identifying high-risk patients who may benefit from more intensive adjuvant therapies, regardless of their response to neoadjuvant systemic therapy.

Few studies have evaluated the prognostic value of the pretreatment tumor burden of involved LNs [19–21]. Of these studies, two studies showed the prognostic value of metabolic tumor volume (MTV), defined as a nodal volume with a standardized uptake value of > 2.5 on ^18^F-FDG PET/CT [20, 21], while another reported the prognostic value of the number of axillary LNs greater than 1 cm in size on CT [19]. However, these studies included patients with clinical or pathologic N0 stage [20, 21] and/or supraclavicular/internal mammary LNs involvement [19, 21]. To the best of our knowledge, the present study is one of the few focusing on the prognostic value of the pretreatment tumor burden of axillary LNs in patients with biopsy-proven axillary LN-positive breast cancer, without the involvement of supraclavicular or internal mammary LNs.

Traditionally, the median or mean has been used as a cutoff point in survival analysis. However, these values are determined entirely by the distribution of variables and are not specific to survival outcomes. Currently, the minimal *p* value approach, which selects the value with the lowest *p* value for survival from the log-rank statistics, is frequently used [22]. However, because the minimal *p*value approach does not reflect the distribution of variables, cutoff points from this approach can be extremely skewed to one side. To address this limitation, we modified the minimal *p* value approach by selecting optimal cutoff points for axillary nodal volume from quantile-based cutoffs (i.e., the median, tertiles, quartiles, and quintiles) with the smallest *p* value for DFS. The 5-year DFS rate decreased as axillary nodal volume increased, with the greatest differences observed at the first tertile (2.6 mL) and fifth quintile (12.0 mL), leading us to classify nodal volumes into three groups: low (< 2.6 mL), intermediate (2.6–12.0 mL), and high (≥ 12.0 mL).

Breast cancer is a heterogeneous disease with prognostic variability depending on the status of biomarkers, including histologic grade, ER, PR, HER2, and Ki-67, even within the same anatomic stage. The introduction of prognostic staging incorporating biomarkers in the eighth edition of the AJCC staging system reflects the complexity of prognosis in breast cancer [23, 24]. In our study, hormone receptor status was a significant prognostic factor for DFS in univariate analysis, and we found that pretreatment nodal volume was significantly larger in patients with ER (−) and PR (−) compared to patients with ER (+) and/or PR (+). The optimal cutoff point for nodal volume was also higher in patients with ER (−) and PR (−) (12.0 mL, fifth quintile), and its clinical significance was more pronounced in this group. Similar to our findings, Yoo et al. reported that the clinical significance of the number of pretreatment axillary LNs greater than 1 cm in size on CT was higher in patients with ER (−) and PR (−) compared to patients with ER (+) and/or PR (+) [19]. These findings suggest that the optimal cutoff point and clinical significance of pretreatment axillary nodal volume may vary depending on the status of biomarkers, and further studies with larger patient cohorts and longer follow-up periods are needed.

There were several limitations to our study. First, the retrospective nature of this study inherently introduces selection bias. Additionally, the exclusion of patients without information about adjuvant radiotherapy or who did not receive pretreatment MRI assessment (*n* = 58) may limit the generalizability of our findings, as MRI is not always part of the standard preoperative imaging in all institutions. Second, this study was conducted at a single institution, which may restrict its external applicability. Multicenter validation would be necessary to confirm the robustness of our findings. Third, we used various types of MRI scanner with different magnetic strengths and slice thicknesses, which may have introduced variability in measurements. Differences in signal-to-noise ratio and spatial resolution between 1.5T and 3T MRI could potentially influence nodal volume estimation, and thicker slice thickness may introduce partial volume effects. A recent prospective study comparing 1.5T and 3T breast MRI found that, when optimized protocols are used, both field strengths provide comparable diagnostic accuracy [25]. However, the impact of magnetic strength on volumetric measurements, particularly for nodal volume assessment, has not been well established. Further studies with standardized MRI protocols are needed to minimize the potential source of variability. Fourth, due to the small number of events, it was difficult to evaluate the clinical significance of pretreatment axillary nodal volume in subgroups with a detailed molecular subtype other than hormone receptor status. Given that axillary tumor burden and response to neoadjuvant systemic therapy vary widely among these subtypes, further exploration would be beneficial to enhance personalized treatment strategies. Fifth, axillary nodal volume assessment was performed by a single radiation oncologist, which may raise concerns about the reliability and reproducibility of the measurements. To address potential variability in manual measurements, further studies should include multiple observers to assess interobserver variability and enhance the reliability of nodal volume as a prognostic tool. Additionally, the use of artificial intelligence–based automated segmentation tools could help improve reproducibility and reduce measurement subjectivity. Finally, while the identified nodal volume cutoffs (2.6 mL and 12.0 mL) were statistically significant, their clinical applicability remains to be validated. Prospective studies utilizing external datasets are needed to confirm these cutoffs and determine their potential role in clinical practice. Despite these limitations, our present study is one of the few to analyze the prognostic value of the pretreatment tumor burden of axillary LNs in patients with breast cancer who have undergone neoadjuvant systemic therapy. As less invasive axillary surgeries become increasingly common, pretreatment nodal volume could potentially serve as a useful tool in risk stratification, helping to guide decisions regarding the need for more intensive adjuvant therapy for high-risk patients, regardless of their posttreatment response.

## 5. Conclusion

In axillary LN-positive breast cancer treated with neoadjuvant systemic therapy, larger nodal volume was associated with poor survival outcomes. Further studies are needed to investigate the role of pretreatment axillary nodal volume for identifying patients who need intensified treatment.

## Figures and Tables

**Table 1:** Patient characteristics.

Characteristics	No. (%)
*Age (years)*	
Median (range)	47 (26–75)

*cT stage (AJCC*8^*th*^)	
cT1/cT2	36 (11.9)/266 (88.1)

*cN stage (AJCC*8^*th*^)	
cN1/cN2a/cN3a	248 (82.1)/26 (8.6)/28 (9.3)

*Highest level of involved axillary LNs*	
I/II/III	176 (58.3)/98 (32.5)/28 (9.3)

*Volume of axillary LNs (cm* ^3^)	
Median (range)	4.2 (0.2–134.2)

*Pathology*	
Invasive ductal carcinoma/others^∗^	299 (99.0)/3 (1.0)

*Histologic grade*	
I/II/III	4 (1.3)/192 (63.6)/98 (32.5)
Unknown	8 (2.6)

*Hormone receptor status*	
ER (+) and/or PR (+)	179 (59.3)
ER (−) and PR (−)	121 (40.1)
Unknown	2 (0.7)

*HER2 status*	
HER2 (+)	103 (34.1)
HER2 (−)	193 (63.9)
Unknown	6 (2.0)

*Ki-67*	
< 20%	62 (20.5)
≥ 20%	206 (68.2)
Unknown	34 (11.3)

*Breast surgery*	
Breast conserving surgery/mastectomy	184 (60.9)/118 (39.1)

*Axillary surgery*	
Sentinel node biopsy/axillary LN dissection	101 (33.4)/201 (66.6)

*ypT stage (AJCC*8^*th*^)	
ypT0/ypTis/ypT1/ypT2/ypT3	41 (13.6)/28 (9.3)/145 (48.0)/83 (27.5)/5 (1.7)

*ypN stage (AJCC*8^*th*^)	
ypN0/ypN1mi/ypN1a/ypN2a/ypN3a	114 (37.7)/39 (12.9)/95 (31.5)/38 (12.6)/16 (5.3)

*Adjuvant radiotherapy*	
Yes/no	233 (77.2)/69 (22.8)

*Adjuvant chemotherapy*	
Yes/no	40 (13.2)/262 (86.8)

*Adjuvant HTx*	
Yes/no	191 (63.2)/111 (36.8)

*Adjuvant targeted therapy*	
Yes/no	115 (38.1)/187 (61.9)

Abbreviations: AJCC, American Joint Committee on Cancer; ER, estrogen receptor; HER2, human epidermal growth factor receptor 2; HTx, hormone therapy; LN, lymph node; PR, progesterone receptor.

^∗^Invasive mammary carcinoma in two patients and metaplastic carcinoma in one patient.

**Table 2:** Prognostic factors for disease-free survival and overall survival rates.

Factors	Disease-free survival				Overall survival			
	Univariate analysis		Multivariate analysis		Univariate analysis		Multivariate analysis	
	Hazard ratio (95% CI)	*p* value	Hazard ratio (95% CI)	*p* value	Hazard ratio (95% CI)	*p* value	Hazard ratio (95% CI)	*p* value
Age (vs. ≥ 35 years)	2.360 (1.215–4.585)	0.011	2.550 (1.292–5.033)	0.007	1.689 (0.579–4.929)	0.337	—	—
cT stage (vs. cT1)	1.701 (0.614–4.711)	0.307	—	—	2.927 (0.395–21.681)	0.293	—	—
cN stage		0.780		0.549		0.140		0.909
cN2a (vs. cN1)	1.081 (0.428–2.733)	0.868	0.725 (0.254–2.074)	0.549	1.041 (0.241–4.496)	0.957	0.708 (0.150–3.345)	0.662
cN3a (vs. cN1)	1.356 (0.577–3.187)	0.484	—	—	2.704 (1.004–7.285)	0.049	0.946 (0.235–3.813)	0.938
Level of involved axillary LNs		0.086		0.313		0.042		0.657
Level II (vs. Level I)	1.854 (1.053–3.266)	0.032	1.536 (0.832–2.837)	0.170	2.186 (0.903–5.291)	0.083	1.615 (0.575–4.532)	0.363
Level III (vs. Level I)	1.749 (0.715–4.279)	0.221	0.912 (0.334–2.892)	0.974	3.838 (1.286–11.455)	0.016	1.312 (0.268–6.058)	0.760
Volume of axillary LNs		0.024		0.032		0.079		0.028
2.6–12 mL (vs. < 2.6 mL)	2.161 (1.050–4.450)	0.036	2.132 (1.025–4.434)	0.043	2.128 (0.686–6.603)	0.191	2.421 (0.655–8.943)	0.185
≥ 12 mL (vs. < 2.6 mL)	2.982 (1.353–6.573)	0.007	3.098 (1.323–7.257)	0.009	3.787 (1.165–12.308)	0.027	5.408 (1.463–19.993)	0.011
Histologic grade (vs. I–II)	1.293 (0.736–2.270)	0.372		—	1.692 (0.770–3.717)	0.190	—	—
Hormone receptor status (vs. positive)	2.010 (1.175–3.439)	0.011	1.354 (0.310–5.921)	0.687	2.207 (0.991–4.915)	0.053	1.626 (0.648–4.085)	0.301
HER2 status (vs. positive)	1.353 (0.751–2.436)	0.314		—	1.178 (0.503–2.758)	0.706	—	—
Ki-67 (vs. < 20%)	1.114 (0.552–2.246)	0.763		—	5.842 (0.783–43.561)	0.085	4.053 (0.527–31.195)	0.179
Breast surgery (vs. breast-conserving surgery)	1.755 (1.029–2.994)	0.039	0.998 (0.470–2.119)	0.996	1.480 (0.675–3.245)	0.327	—	—
Axilla surgery (vs. sentinel node biopsy)	1.117 (0.622–2.007)	0.711		—	0.906 (0.388–2.118)	0.820	—	—
ypT stage		0.126		0.008		0.329		—
ypTis, ypT1 (vs. ypT0)	1.801 (0.633–5.123)	0.270	3.317 (1.135–9.692)	0.028	3.608 (0.477–27.319)	0.214	—	—
ypT2-3 (vs. ypT0)	2.707 (0.929–7.891)	0.068	5.724 (1.815–16.809)	0.003	4.699 (0.595–37.111)	0.142	—	—
ypN stage		0.821		0.941		0.401		0.417
ypN1mi, ypN1a (vs. ypN0)	1.142 (0.629–2.075)	0.662	0.927 (0.486–1.769)	0.818	1.248 (0.502–3.106)	0.633	1.395 (0.510–3.816)	0.516
ypN2a (vs. ypN0)	0.956 (0.381–2.396)	0.923	0.751 (0.267–2.109)	0.587	1.374 (0.362–5.220)	0.641	1.072 (0.219–5.256)	0.932
ypN3a (vs. ypN0)	1.616 (0.550–4.752)	0.383	1.083 (0.310–3.784)	0.900	3.160 (0.838–11.920)	0.089	3.245 (0.796–13.229)	0.101
Adjuvant radiotherapy (vs. yes)	1.845 (1.047–3.251)	0.034	2.630 (1.425–4.857)	0.002	1.318 (0.550–3.160)	0.535	—	—
Adjuvant chemotherapy (vs. yes)	0.593 (0.298–1.180)	0.137		—	0.534 (0.199–1.433)	0.213	—	—
Adjuvant HTx (vs. yes)	2.017 (1.183–3.442)	0.010	2.695 (1.501–4.840)	0.001	2.161 (0.981–4.762)	0.056	0.723 (0.090–5.821)	0.761
Adjuvant targeted therapy (vs. yes)	1.620 (0.901–2.911)	0.107		—	1.228 (0.541–2.788)	0.624	—	—

Abbreviation: CI, confidence interval.

## Data Availability

The data that support the findings of this study are available on request from the corresponding author. The data are not publicly available due to institution's policy.

## Supporting Information

Supporting Figure 1 shows disease-free survival rates by quantile-based cutoffs for pretreatment axillary nodal volume.
