# Supplementary material for: Pretreatment Axillary Nodal Volume as a Prognostic Factor for Breast Cancer
Source: Breast J. 2025 Apr 23;2025:1823771. doi: 10.1155/tbj/1823771 (PMC12043390; doi:10.1155/tbj/1823771)
Supplement: Supporting Information — Additional supporting information can be found online in the Supporting Information section. [file 1823771.f1.docx]

**Supporting Figure 1.** Disease-free survival rates by quantile-based cutoffs for pretreatment axillary nodal volume: median (a) tertile (b), quartile (c), and quintile (d).
